# Detection and Typing of Norovirus from Frozen Strawberries Involved in a Large-Scale Gastroenteritis Outbreak in Germany

**DOI:** 10.1007/s12560-013-9118-0

**Published:** 2013-07-26

**Authors:** Dietrich Mäde, Katja Trübner, Eckehard Neubert, Marina Höhne, Reimar Johne

**Affiliations:** 1 Department of Food Safety, State Office for Consumer Protection Saxony-Anhalt, Freiimfelder Str. 68, 06112 Halle, Germany; 2 Department 5-Official Food Analysis, Saxon State Laboratory of Health and Veterinary Affairs, Zschopauer Straße 87, 09111 Chemnitz, Germany; 3 Division 15, Consultant Laboratory for Noroviruses, Robert-Koch-Institute, Seestrasse 10, 13353 Berlin, Germany; 4 Federal Institute for Risk Assessment, National Reference Laboratory for Monitoring Bacteriological and Viral Contamination of Bivalve Molluscs, Diedersdorfer Weg 1, 12277 Berlin, Germany

**Keywords:** Norovirus, Strawberries, Genotyping, Real-time RT-PCR, Food safety

## Abstract

During September/October 2012, a norovirus gastroenteritis outbreak affecting about 11,000 people occurred in Germany. Epidemiological studies suggested that frozen strawberries represented the vehicle of infection. We describe here the analysis of frozen strawberries for the presence of norovirus. Samples were taken by applying a stratified subsampling scheme. Two different methods for virus extraction from strawberries were compared. First, viruses were eluted from strawberries under alkaline conditions and concentrated using a polyethylene glycol precipitation. Second, ultrafiltration was applied for concentration of viruses rinsed off of the berries. In both cases, RNA was extracted and analyzed by real-time RT-PCR. Application of the ultrafiltration method generally resulted in a lower detection rate. Noroviruses were detected in 7/11 samples derived from the lot of strawberries implicated in the outbreak using the precipitation method. Typing of norovirus revealed three different genotypes including a combination of norovirus genotype II.16 (viral polymerase) and II.13 (viral capsid). This genotype combination was also found in some of the patients that were involved in the outbreak, but that had not been reported in Germany so far. In conclusion, heterogeneously distributed noroviruses in frozen strawberries can be detected by applying an optimized combination of sampling procedures, virus extraction methods, and real-time RT-PCR protocols. The detection of several different genotypes in the strawberries may suggest contamination from sewage rather than from a single infected food handler.

## Introduction

Norovirus infection is a major cause of human gastroenteritis in the Northern hemisphere. In Germany, about 115,000 laboratory confirmed cases were registered in 2011 which greatly exceeded all other gastro-enteric pathogens (Anonymous 2012a). Although it is difficult to obtain exact numbers, it has been estimated that about 40 % of norovirus infections in the US were of foodborne origin (Mead et al. 1999). Fresh produce and especially soft berries are considered to be food items that are at high risk to be contaminated with norovirus (FAO/WHO 2008). Several outbreaks were recorded, in which contaminated raspberries were identified as the source of infection (Le Guyader et al. 2004; Falkenhorst et al. 2005; Korsager et al. 2005; Cotterelle et al. 2005; Fell et al. 2007; Sarvikivi et al. 2012).

In September/October 2012, an outbreak of norovirus gastroenteritis was recorded in eastern Germany. A total of 390 facilities, mostly schools, located in five Federal States of Germany were affected with 10,950 registered gastroenteritis cases which included 38 hospitalizations. Results of epidemiological and trace-back investigations suggest that a consignment of frozen strawberries imported from China was the source of the outbreak. Case–control studies revealed a strong correlation between gastroenteritis and the consumption of strawberry compote served cold in canteens (Anonymous 2012b). This was the largest food-borne outbreak to have ever been reported in Germany.

Frozen strawberries and a variety of other foods, which were served by the catering company involved in the outbreak, were analyzed for norovirus RNA. The detection of viruses in soft berries is often compromised by low recovery rates and the presence of PCR inhibitors (Schrader et al. 2012). Efficient and reliable virus extraction and concentration procedures, which have to be applied to the samples before molecular detection of viruses, are often laborious and time-consuming. Various protocols for extraction and purification of norovirus from soft berries have been developed including methods based on precipitation of the virus or on ultrafiltration procedures (Dubois et al. 2002; Mäde et al. 2005; Butot et al. 2007). Comparison of both methods using artificially contaminated food samples revealed different results that were mainly dependent on the type of sample and the virus concentration (Scherer et al. 2010). The methodology of norovirus detection in food samples is subject of international standardization (Anonymous 2013).

In this study, two different laboratory methods for the detection of noroviruses were applied to samples of frozen strawberries that were implicated in a large-scale gastroenteritis outbreak. The methods including the sampling strategy are described in detail. Genotyping of the detected noroviruses was performed in order to confirm the link between the contaminated food and patient samples. The study should contribute to the development of reliable methods for detection of noroviruses in food and to improve the identification procedures for sources of food-borne virus infections.

## Materials and Methods

### Sampling

Sampling procedures and transportation steps were carried out by keeping the fruits under frozen conditions. If originally packaged and closed 10 kg boxes were available, each box (hereafter designated as sample) was sampled with three subsamples of approximately 1 kg each, targeting the upper, middle, and lower part of the box. In catering facilities and kitchens, where only opened boxes were available, one or two 1 kg subsamples were taken dependent on the amount of material available. The samples were derived from four different lots (A–D) of frozen strawberries. Only lot A, which comprised 44 tons of frozen strawberries imported from China, was implicated in the norovirus gastroenteritis outbreak. Samples and lots are listed in Table 1. In total, 28 subsamples belonging to 11 samples from lot A, 5 subsamples belonging to 3 samples from lot B, 3 subsamples belonging to 1 sample of lot C, and 3 subsamples belonging to 1 sample of lot D were analyzed. Each subsample was analyzed in duplicate.

**Table 1:** The results of real-time RT-PCR analysis of strawberry samples after application of two different virus concentration procedures

Sample number	Lot	Subsample	Results of precipitation method	Results of ultrafiltration method
1	A	1	+(GI and GII) [GI.9]^a^	+(GII)
2	A	1	+(GII)	+(GII)
		2	+(GII)	–
		3	+(GII)	–
3	A	1	–	–
4	A	1	+(GII)	–
		2	+(GII) [GII.16/II.13]^a/b^	–
5	A	1	–	–
		2	–	–
		3	–	–
6	A	1	–	–
		2	+(GI and GII)	+(GII)
		3	–	–
7	A	1	+(GII)	n.d.
		2	+(GI and GII)	n.d.
		3	–	n.d.
8	A	1	–	n.d.
		2	–	n.d.
		3	–	n.d.
9	A	1	+(GII) [GII.6]^a^	n.d.
		2	+(GII)	n.d.
		3	+(GII)	n.d.
10	A	1	–	n.d.
		2	–	n.d.
		3	–	n.d.
11	A	1	+(GII)	n.d.
		2	+(GII)	n.d.
		3	+(GII)	n.d.
12	B	1	–	–
13	B	1	–	–
14	B	1	–	–
		2	–	–
		3	–	–
15	C	1	–	–
		2	–	–
		3	–	–
16	D	1	–	–
		2	–	–
		3	–	–

n.d. not done

^a^Indicates samples, for which norovirus sequences could be generated by subsequent nucleic acid sequence analysis

^b^Random amplification of RNA was applied to an aliquot of the RNA before nested RT-PCR amplification and sequencing

### Virus Extraction Using the Precipitation Method

A protocol based on alkaline elution and polyethylene glycol (PEG) precipitation was used as previously described (Dubois et al. 2002; Butot et al. 2007). The procedure was modified by extending the originally described PEG precipitation at 4 °C step to an overnight incubation. In brief, 25–30 g of frozen strawberries were transferred into a mesh filter bag, and 40 mL of TRIS glycine beef extract buffer (TGBE, pH 9.5) as well as 1,140 units of pectinase from *Aspergillus aculeatus* (Sigma, Deisenhofen, Germany) were added. To each of the samples, 1 μL of bacteriophage MS2, corresponding to 100,000 plaque-forming units, was added as process control. In each series of extraction experiments, a negative process control using TGBE and MS2 only was analyzed together with the samples. The thawed fruits were smashed manually in the buffer and incubated on a rocking platform at room temperature with constant rocking at approximately 300 rpm for 20 min. The pH was checked after 20 min and adjusted to pH 9.5 using 12.5 n NaOH solution. Thereafter, the incubation was continued for 10 min and the pH was checked and adjusted again. In some cases, the process had to be repeated in order to obtain pH 9.5. The eluate from the filtered compartment was transferred into a 50 mL tube and clarified by centrifugation at 4,500×*g* for 60 min at 5 °C. The clear supernatant was decanted into a round bottom centrifuge tube and the pH was now adjusted to pH 7.0 with 10 n HCl. After the addition of 0.25 volumes of a 5 × PEG/NaCl solution (50 % (w/v) PEG 8000, 1.5 M NaCl), the samples were incubated with constant rocking at 350 rpm at 4 °C overnight and thereafter centrifuged at 10,000×*g* for 30 min at 5 °C. After decanting the supernatant, the pellet was centrifuged again at 10,000×*g* for 5 min at 5 °C and the supernatant was carefully removed by pipetting. The gelatinous pellet was transferred into a 2-mL reaction tube using a sterile glass rod. Remaining parts of the pellet in the centrifuge tube and on the glass rod were removed by adding 500 μL PBS and transferred into the same reaction tube. After homogenization, 500 μL chloroform–butanol (1:1 v/v) was added, thoroughly mixed and then incubated at room temperature for 5 min. The aqueous phase (500 μL) was collected after centrifugation at 10,000×*g* for 15 min, transferred to a 15-mL Falcon tube and subjected to RNA extraction.

### Virus Extraction Using the Ultrafiltration Method

For the ultrafiltration method, a modified protocol according to Mäde et al. (2005) was applied. A total of 15 g of frozen strawberries was transferred into a 15-mL reaction tube and rinsed quickly with 25 mL of ice-cold PBS until the color changed slightly into red (modification by Mormann S, and Becker B, personal communication 2011). After decanting the PBS, 1 μL of phage MS2, corresponding to 100,000 plaque-forming units, was added as process control. A negative process control using PBS and MS2 only was analyzed together with the samples. The samples were centrifuged at 3,000×*g* for 10 min. The supernatants were filtered sequentially through 0.45 and 0.2 μm syringe filters (Whatman, Dassel, Germany) and transferred into Vivaspin 50,000 MWCO concentrators (Sartorius, Göttingen, Germany). Thereafter, the samples were centrifuged at 4,000×*g* at 4 °C for 30 min to 4 h until a final volume of 500 μL was obtained. These were subsequently used for RNA extraction.

### RNA Extraction

RNA was extracted using the Viral RNA mini Kit (Qiagen, Hilden, Germany). The manufacturer’s protocol was up-scaled to 500 μL starting material using the amount of 2,000 μL AVL buffer and the same amount of ethanol. The resulting solution was loaded onto a QIAamp Mini column in stepwise portions of 630 μL each. The samples were washed with buffers AW1 and AW2 and finally eluted using 60 μL of buffer AVE. An additional negative extraction control consisting of 0.1× TE buffer and 1 μL MS2 (100,000 pfu) was included in each series of experiments.

### Real-Time RT-PCR

Each portion of RNA extracted was subjected at least twice to real-time RT-PCRs for the detection of norovirus genogroups GI, GII, and bacteriophage MS2 with primers and probes as described previously (Dreier et al. 2005; Anonymous 2012c; da Silva et al. 2007; Svraka et al. 2007; Loisy et al. 2005; Kageyama et al. 2003). The primer and probe combinations are shown in Table 2. 10 and 2 μL of template RNA were analyzed in 50 μL reaction mixes in parallel. The virus extraction procedures were monitored using the bacteriophage MS2 as a process control and estimating the recovery rate by comparison of the MS2-specific ct values derived from the samples to that of the original MS2 solution (Dreier et al. 2005). Samples with insufficient recovery of MS2 resulting in a ct-value of 35 or higher were reanalyzed using 1 μL of template. This procedure meets the requirements set in ISO/TS 15216-2:2013 demanding the application of a diluted RNA extract to monitor inhibitory effects (Anonymous 2013). The assay targeting norovirus GI was set up as single reaction containing 25 μL 2× QuantiTect Probe RT-PCR Master Mix, 0.5 μL QuantiTect RT Mix (Qiagen Hilden, Germany), 900 nmol/L each primer QNIF4 and NV1LCR, 250 nmol/L probe NVGG1p, template RNA (10, 2, and 1 μL, respectively), and nuclease-free water up to 50 μL. Norovirus GII and the process control virus MS2 were analyzed as duplex real-time PCR using 25 μL 2× QuantiTect Probe RT-PCR Master Mix, 0.5 μL QuantiTect RT Mix (Qiagen Hilden, Germany), 900 nmol/L each primer QNIF2 and COG2R, 250 nmol/L probe QNIFS, 200 nmol/L primer MS2-TM2-F, 700 nmol/L primer MS2-TM2-R, 200 nmol/L probe MS2-TM2, template RNA (10 μL, 2 μL, and 1 μL respectively), and nuclease free water up to 50 μL. Real-time RT-PCR was performed in a CFX 96 (Biorad, München, Germany) and a LightCycler 480 (Roche Diagnostics, Mannheim, Germany) instrument as follows: 50 °C for 30 min, 95 °C for 15 min, and then 50 cycles of 95 °C for 15 s, 56 °C for 60 s, and 72 °C for 10 s.

**Table 2:** Oligonucleotides used in the real-time RT-PCR assays

Assay	Primer	Sequence (5′–3′)	Working concentration (nmol/L)	References
Norovirus GI	QNIF4	CGC TGG ATG CGN TTC CAT	900	da Silva et al. (2007)
	NV1LCR	CCT TAG ACG CCA TCA TCA TTT AC	900	Svraka et al. (2007)
	NVGG1p	(FAM)-TGG ACA GGA GAY CGC RAT CT-(TAMRA)	250	Svraka et al. (2007)
Norovirus GII and MS2 (duplex)	QNIF2	ATG TTC AGR TGG ATG AGR TTC TCW GA	900	Loisy et al. (2005)
	COG2R	TCG ACG CCA TCT TCA TTC ACA	900	Kageyama et al. (2003)
	QNIFS	(FAM)-AGC ACG TGG GAG GGC GAT CG-(TAMRA)	250	Loisy et al. (2005)
	MS2-TM2-F	GGC TGC TCG CGG ATA CCC	200	Dreier et al. (2005)
	MS2-TM2-R	TGA GGG AAT GTG GGA ACC G	700	Dreier et al. (2005)
	MS2-TM2	(JOE)-ACC TCG GGT TTC CGT CTT GCT CGT-(BBQ)	200	Dreier et al. (2005)

Y = C/T; R = A/G; W = A/T

### Random Amplification of RNA

In order to multiply the nucleic acids before specific amplification, random amplification using the Whole Transcriptome Amplification (WTA) 2 kit (Sigma, Deisenhofen, Germany) was applied to one RNA extract derived from sample 4. A total of 5 μL of RNA extracted from the subsample was amplified according to the manufacturer’s protocol; 13 amplification cycles were most efficient, as demonstrated by norovirus-specific real-time PCR of the WTA-products.

### Nested RT-PCR, DNA Sequencing, and Genotyping

For the sequencing of norovirus positive RNA preparations and randomly amplified RNA, amplicons of 338 bp from the RNA-dependent RNA polymerase gene (ORF1, region A) and of 259 bp from the junction region between ORF1 and ORF2 (region C) were generated by nested RT-PCR and analyzed. Sample processing, amplification, and sequencing of region A were performed as described by Oh et al. (2003). Primers for amplification and sequencing of region C were used as described by Bernard et al. (2013). PCR products were sequenced directly using the BigDye terminator cycle sequencing kit and an ABI 3500xLDx Genetic Analyzer (Applied Biosystems, Darmstadt, Germany). Sequences were aligned to prototype sequences drawn from GenBank using CLUSTAL W, version 1.6, and the neighbor joining and DNADIST program of the Phylogeny Interference Package (PHYLIP), version 3.57c (Felsenstein 1989).

## Results

### Detection of Norovirus in Strawberry Samples

Samples of frozen strawberries from lot A implicated in the gastroenteritis outbreak and lots B–D, which were received by the laboratory for control purposes, were analyzed using the precipitation method and the ultrafiltration method in parallel. The results of the analyses are summarized in Table 1. Norovirus RNA was only detected in samples derived from the consignment involved in the gastroenteritis outbreak. In general, the precipitation method showed a higher norovirus detection rate as compared to the ultrafiltration method. Using the ultrafiltration method, only one replicate per subsample tested norovirus positive in samples 1, 2, and 6. In one case (sample 4), the sample could be only tested norovirus positive by use of the precipitation method, whereas both subsamples tested with the ultrafiltration method remained norovirus negative. In addition, only RNA derived from the precipitation method could be successfully amplified in the nested RT-PCRs used for genotyping (see below). As increasing evidence for a higher sensitivity of the precipitation method arose during the application of the method in the outbreak, samples 7–11 were further analyzed by the precipitation method only.

The extraction efficiencies of the procedures were monitored by adding the bacteriophage MS2 and estimating its recovery rate. When using 10-μL RNA template, these recovery rates varied between 0.1 % and lower, and increased up to 1 % in some of the samples using 2 and 1 μL RNA template. The MS2 recovery rates were largely similar when comparing the precipitation method to the ultrafiltration method despite the mentioned differences in the norovirus detection rates. Moreover, in some of the samples without recovery of MS2, norovirus RNA was detected (data not shown).

### Genotyping of the Norovirus RNA

Attempts to sequence fragments of the norovirus genome present in the strawberries were successful at first for sample 4. Using RNA extracted from that sample, a 295 bp long sequence of the polymerase-encoding region (GenBank acc.-no. KC207117) could be genotyped as GII.16. However, amplification of a nested RT-PCR product from the ORF1/ORF2 junction region was not successfully using RNA of sample 4. In order to increase the amount of nucleic acids in this sample, a random amplification of RNA was performed by application of the WTA2 kit to the RNA of sample 4. Using this randomly amplified nucleic acids, a 228 bp long sequence of the ORF1/ORF2 junction (viral capsid, GenBank acc.-no. KC207118) could be amplified and identified as genotype II.13. This combination of genotypes II.16 (viral polymerase) and II.13 (viral capsid) was also detected in some of the stool samples derived from patients infected during this outbreak (data not shown) where it was shown to be a recombinant virus generated from both genotypes. Comparison of the ORF1/ORF2 junction between these patients and strawberries revealed 99.5–100 % nucleotide sequence identity. In addition, short sequences generated later by sequencing of real-time RT-PCR products from samples 1 and 9 could be grouped as genotypes GI.9 and GII.6, respectively. Both genotypes were detected in some of the human samples as well.

## Discussion

In total, 7 out of 11 samples of lot A, derived from three different canteens and four original boxes taken at the warehouse, tested positive for norovirus GI and GII and the results were subsequently reported to the Rapid Alert System for Food and Feed (RASFF Portal 2012a). One of the samples (sample 4) was analyzed independently by two different laboratories. In both laboratories a norovirus positive result was obtained by application of the precipitation method. However, norovirus could not be detected in all of the samples taken from lot A. This might be due to low norovirus concentrations combined with significant amounts of PCR inhibitors present in berries (Schrader et al. 2012). Heterogeneous norovirus contamination in the consignment, which consisted of 44 tons, should also be taken into consideration. Large lots need to be addressed using the appropriate sampling strategies (Petersen and Esbensen 2005). If available, three subsamples were taken from each box of 10 kg and analyzed in duplicate. This number of subsamples is considered to be appropriate for a reliable analysis. If only a small number of samples is analyzed, false negative results cannot be excluded due to the lot heterogeneity.

As the detection of viruses in berries is generally very difficult (Scherer et al. 2010, 2012), the performance of the applied methods was monitored by adding defined amounts of the bacteriophage MS2 to the samples and measuring the detectable amount after the processing of the sample. The resulting recovery rates were generally very low ranging from 0 to 1 %. The MS2 recovery rate is influenced by several factors including the efficiencies of the virus elution from the berries, the virus concentration, and the RNA extraction. In addition, inhibitory substances present in the final RNA solution negatively influence the MS2 recovery rate. In some of the cases described here, a decrease in the amount of sample RNA in the real-time RT-PCR resulted in an increased recovery rate. This suggests the presence of inhibitory substances, which were diluted by using a smaller volume of the RNA per 50 μL reaction mixture. Testing of different dilutions of sample RNA is commonly used to minimize PCR inhibition (Schrader et al. 2012) and is also a requirement of the ISO/TS 15216:2 method (Anonymous 2013). As the low concentration of norovirus RNA and the presence of PCR inhibitors adversely affect the results, strawberry samples should be generally analyzed using different amounts of template.

The methodology applied could successfully demonstrate the presence of norovirus in frozen strawberries implicated in large-scale gastroenteritis outbreaks. The precipitation method showed a higher norovirus detection rate as compared to the ultrafiltration method; therefore, this method should be used for testing of strawberry samples in the future. It has been reported that the precipitation method performs better than the ultrafiltration method when examining artificially contaminated raspberries (Scherer et al. 2010). Consequently, a protocol for the precipitation method was made available online just recently (Anonymous 2012c), and published as the international technical specification ISO/TS 15216-2:2013 (Anonymous 2013).

Reliable genotyping of norovirus detected in the strawberries was only successful in one subsample. This may be because RNA fragments that are long enough and at concentrations high enough in the solution are required for successful amplification and sequencing. Amplification of the ORF1/ORF2 junction from sample 4, was only successful with the additional inclusion of a random amplification step, which may be an option in the future for a general treatment of RNA derived from berries prior to RT-PCR amplification. The detected recombinant genotype II.16/II.13 has never been reported before in Germany as far as the authors know. However, a genotype II.16/II.13 recombinant reference strain from Kolkata, India, was deposited recently in the GenBank database (acc.-no. AB592965). Based on the high sequence identities between norovirus detected in the strawberries and in some of the outbreak cases, and the fact that this strain has not been described in Germany so far, the contaminated imported strawberries can be assumed to be the cause of this outbreak. In addition to this sample, the real-time PCR products from two subsamples were successfully sequenced; however, the very short lengths of these sequences demand a careful interpretation of the genotype assignment in these cases.

The laboratory investigations confirm the results of the epidemiological investigations, which identified strawberries as the possible cause of the outbreak (Anonymous 2012b). Recently, contaminated strawberries have also been reported as a source of norovirus outbreaks in the United States (Hall et al. 2012). Although the distinct contamination source in most cases is not known, irrigation water contaminated with human sewage has been repeatedly considered as a cause of virus contamination in berries (FAO/WHO 2008; EFSA 2011). This route of virus contamination has also been demonstrated experimentally (Brassard et al. 2012). Under this scenario, contamination with a mixture of different viruses can be expected. The detected variety of norovirus genotypes in the strawberries investigated here would be in accordance with this hypothesis. In order to prevent such contaminations, the *Codex Alimentarius* Committee published guidelines on the application of general principles of food hygiene to the control of viruses in food including berries in 2012. Food business operators need to be aware of the risk of contamination of soft berries. Virus contamination can be generally prevented by proper application of hygienic principles at all stages of production (CAC/GL 79-2012, 2013).

The increasing lot size of fresh produce, nationwide operating caterers and the globalization of primary production require new tools to address food safety. Special attention needs to be focused on the local conditions of primary production in the countries of origin, especially in areas where clean water is limited (FAO/WHO 2008; EFSA Panel on Biological Hazards (BIOHAZ) 2011; CAC/GL 79-2012, 2013). In addition, the testing of food, which is frequently implicated in norovirus gastroenteritis outbreaks, should be considered upon importation. The findings of the investigations described here, and the detection of hepatitis A virus in imported frozen strawberries several weeks later (RASFF Portal 2012b), triggered an amendment of import regulations in the European Union.
